# Near-Complete SARS-CoV-2 Seroprevalence among Rural and Urban Kenyans despite Significant Vaccine Hesitancy and Refusal

**DOI:** 10.3390/vaccines11010068

**Published:** 2022-12-28

**Authors:** Carolyne Nasimiyu, Isaac Ngere, Jeanette Dawa, Patrick Amoth, Ouma Oluga, Carol Ngunu, Harriet Mirieri, John Gachohi, Moshe Dayan, Nzisa Liku, Ruth Njoroge, Raymond Odinoh, Samuel Owaka, Samoel A. Khamadi, Samson L. Konongoi, Sudi Galo, Linet Elamenya, Marianne Mureithi, Omu Anzala, Robert Breiman, Eric Osoro, M. Kariuki Njenga

**Affiliations:** 1 Global Health Program, Washington State University (WSU), Nairobi 00100, Kenya; 2 Paul G. Allen School of Global Health, Washington State University (WSU), Pullman, WA 99163, USA; 3 KAVI-Institute for Clinical Research, University of Nairobi, Nairobi 00202, Kenya; 4 Directorate of Public Health, Kenya Ministry of Health, Nairobi 00100, Kenya; 5 Directorate of Health, Nairobi Metropolitan Services, Nairobi 00100, Kenya; 6 School of Public Health, Jomo Kenyatta University of Agriculture and Technology, Nairobi P.O. Box 62000-00200, Kenya; 7 Center for Virus Research, Kenya Medical Research Institute, Nairobi 00200, Kenya; 8 Department of Health Services, County Government of Kakamega, Kakamega 50100, Kenya; 9 Hubert Department of Global Health, Rollins School of Public Health, Emory University, Atlanta, GA 30322, USA

**Keywords:** COVID-19 vaccine, vaccine hesitancy, vaccine refusal, herd immunity, SARS-CoV-2 seroprevalence

## Abstract

Considering the early inequity in global COVID-19 vaccine distribution, we compared the level of population immunity to SARS-CoV-2 with vaccine uptake and refusal between rural and urban Kenya two years after the pandemic onset. A population-based seroprevalence study was conducted in the city of Nairobi (n = 781) and a rural western county (n = 810) between January and February 2022. The overall SARS-CoV-2 seroprevalence was 90.2% (95% CI, 88.6–91.2%), including 96.7% (95% CI, 95.2–97.9%) among urban and 83.6% (95% CI, 80.6–86.0%) among rural populations. A comparison of immunity profiles showed that >50% of the rural population were strongly immunoreactive compared to <20% of the urban population, suggesting more recent infections or vaccinations in the rural population. More than 45% of the vaccine-eligible (≥18 years old) persons had not taken a single dose of the vaccine (hesitancy), including 47.6% and 46.9% of urban and rural participants, respectively. Vaccine refusal was reported in 19.6% of urban and 15.6% of rural participants, attributed to concern about vaccine safety (>75%), inadequate information (26%), and concern about vaccine effectiveness (9%). Less than 2% of vaccine refusers cited religious or cultural beliefs. These findings indicate that despite vaccine inequity, hesitancy, and refusal, herd immunity had been achieved in Kenya and likely other African countries by early 2022, with natural infections likely contributing to most of this immunity. However, vaccine campaigns should be sustained due to the need for repeat boosters associated with waning of SARS-CoV-2 immunity and emergence of immune-evading virus variants.

## 1. Introduction

The COVID-19 vaccine has replaced the earlier mitigation measures as the most effective measure for controlling the spread of the SARS-CoV-2 virus and ending the public health and economic devastations brought about by the most severe pandemic since the 1918 influenza pandemic [1]. In early 2021, the World Health Organization (WHO) advocated for scaling up vaccination to achieve the herd immunity target of vaccinating at least 70% of the population in each country globally by June 2022 [2]. Whereas vaccine inequity was experienced early, global vaccine production reached nearly 1.5 billion monthly doses in November 2021, providing enough vaccines to achieve equitable distribution [3]. For Africa, there was a steady increase in vaccine deliveries through the COVID-19 Vaccine Global Access (COVAX), starting from February 2021 and reaching the optimal target numbers between November 2021 and January 2022 [4]. By the end of January 2022, COVAX had delivered >30% of the 1.6 billion doses required to vaccinate 70% of the population in the continent [5]. 

With vaccine availability addressed, Africa embarked on vaccinating at least 36 million eligible persons weekly from January 2022 to achieve the 70% coverage global target by June 2022 [6]. As of May 2022, the WHO reported that only 14.7% of the population in the African region had been fully vaccinated against COVID-19 [7]. This low coverage was attributed to multiple factors, including late realization of vaccine equity, slow roll-out of country vaccination programs, and high levels of vaccine hesitancy and refusal [5]. Lack of confidence in safety and effectiveness and inadequate knowledge are the most common causes of vaccine hesitancy and refusal [8,9]. There have been wide variations in levels of hesitancy globally, ranging from 17% to 60% in Europe, 9–17% in North America, and 2–36% in Africa [10,11,12]. On the other hand, there has been minimal data from Africa on COVID-19 vaccine refusal, often underpinned by strong cultural, religious, political, and emotional factors that can be difficult to overcome [13]. 

In March 2021, one year after reporting its first COVID-19 case, Kenya rolled out the COVID-19 vaccination program, initially limiting it to frontline health workers, other essential services providers, and security personnel [14]. While the program was gradually expanded, it was not until November 2021 that the country launched a national vaccination program targeting 35.5 million persons aged ≥ 15 years old [15]. While COVID-19 vaccines were widely available countrywide by the end of December 2021, only 17.6% of the population was fully vaccinated by the end of February 2022. Prior studies in Kenya had shown >34% immunity eight months after introduction of SARS-CoV-2 in the country (November 2020), and modeling had forecasted achieving herd immunity by the end of 2021 [16,17]. Kenya’s capital city, Nairobi, was the hotspot throughout the early phase of the pandemic, while the rural areas often accounted for less than 50% of COVID-19 cases reported [15]. Here, we determined the levels of SARS-CoV-2 population immunity in urban and rural Kenya two years into the pandemic, and evaluated vaccine hesitancy and refusal in the two populations. 

## 2. Materials and Methods

### 2.1. Study Site and Design

**Figure 1:**
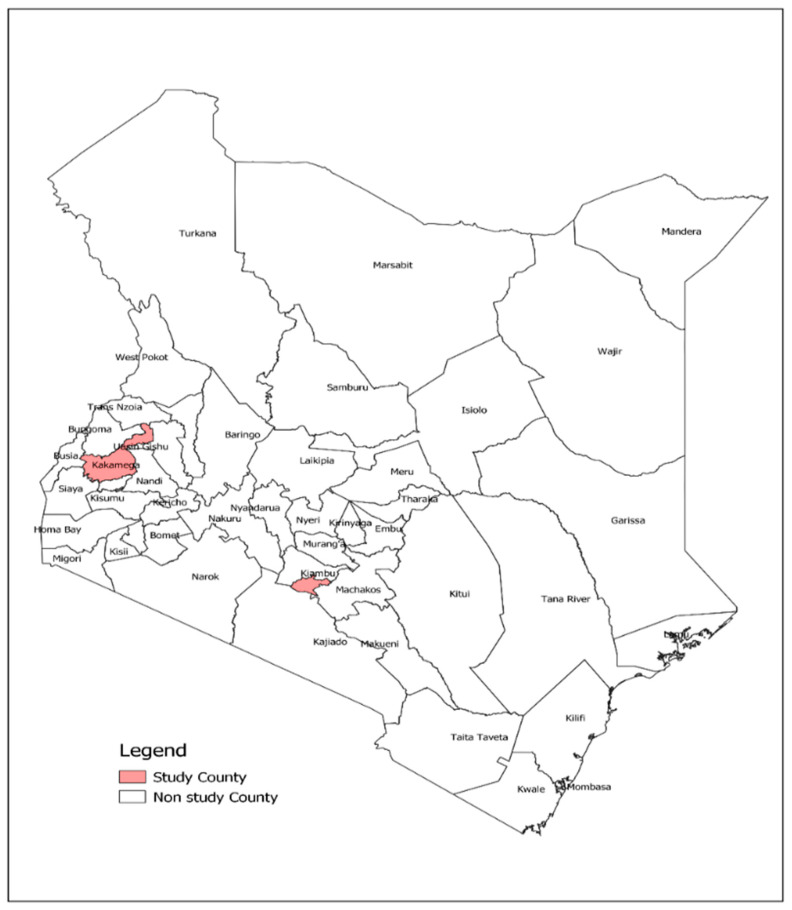
Map of Kenya showing the location of the two study sites: Nairobi, the capital city, and Kakamega, the rural site.

We conducted a cross-sectional survey among residents of urban (Nairobi city) and rural (Kakamega) counties of Kenya (Figure 1) between 17 January and 24 February 2022, 10 months after the COVID-19 vaccine introduction and 2 years after the first COVID-19 case was detected in the country. Nairobi county, which is the most populous urban area in the country, had been a hotspot of COVID-19 transmission throughout the pandemic, whereas Kakamega county, located 350 kilometers west of Nairobi, is 90% rural [16] and was never classified as a hotspot throughout the pandemic. Nairobi was included as a high-transmission county while Kakamega county was randomly selected from 37 low-transmission counties. 

### 2.2. Participant Selection

The sample size was estimated at 768 persons per site as determined using the Fleiss formula [18] with an assumption of a 35–50% level of SARS-CoV-2 prevalence [16] and 5% precision. Further estimating an average of 3 persons per household, the number of households was determined as 256. At each study site, a three-stage random sampling was conducted, first to select the administrative sub-counties, second the wards where the household survey would be rolled out, and third, the households. Wards are the smallest administrative units within the counties. In each county, we randomly selected 30–50% of the wards for inclusion into the study. To identify households in the wards, random geographic coordinates corresponding to 256 households were generated. These households were distributed across the wards proportionate to the total population in the ward. All occupants within the selected households were eligible for enrolment irrespective of age.

### 2.3. Procedure for Replacement of Selected Households

Criteria for replacing selected households included households without a suitable respondent to grant consent, households whose head declined to grant consent, and if there was no household at the expected geocoordinates. Households without a competent adult or household head were revisited twice before being declared non-respondent. Replacement was carried out using randomly selected replacement households. Participants who were not home during the study visit were revisited within seven days during a time they were likely to be available.

### 2.4. Data Collection

Data were collected from consenting participants using a structured questionnaire (Table S1), programmed on the Research Electronic Data Capture (REDCap) tool [19] and stored in Washington State University (WSU) servers. The data elements in the questionnaire included sociodemographic data (age, sex, education level, occupation), past medical history (COVID-19 diagnosis, underlying chronic diseases such as hypertension, diabetes, heart disease, cancer, etc.), COVID-19 knowledge and practices, and COVID-19 vaccine awareness and acceptability. The questions were adopted from previous surveys conducted by the Kenya Ministry of Health, Trends, and Insights for Africa (TIFA) and published local studies [16,20,21]. The data tools were pretested, piloted, and customized for the Kenyan context. The COVID-19 knowledge and attitudes questionnaire was administered to participants aged ≥ 12 years, while the vaccine awareness and acceptability questions were administered to participants aged ≥ 18 years. The questionnaires were translated to Kiswahili, the national language, and where necessary were administered in local languages. The questionnaires were administered in-person by trained research assistants.

### 2.5. Serum Collection and Testing for SARS-CoV-2 IgG Antibodies

Venous blood samples (approximately 5 mL for adults and children aged 13 or older, 2–3 mL for children 2–12 years, and 1.5 mL for children <2 years) for antibody assays were collected from each participant. The blood samples were collected using a sterile technique and shipped at 2–8 °C to the Center for Virus Research at the Kenya Medical Research Institute (CVR/KEMRI) in Nairobi, Kenya, for serum separation, testing, and storage. To detect both infection- and vaccine-induced SARS-CoV-2 spike protein antibodies, the Wantai SARS-CoV-2 total antibodies ELISA kit (Wantai Biological Pharmacy, Beijing, China) was used, while including extra validation steps previously described [16]. 

### 2.6. Statistical Analysis

Data were cleaned and analyzed using R Statistical Software, version 4.2.0 [22]. Descriptive statistics were determined for sociodemographic information, history of chronic illness or COVID-19 illness, knowledge and attitudes on COVID-19 vaccines, source of information on COVID-19 vaccines, and vaccine uptake. Where applicable, categorical variables were compared using the chi-square test and Fisher’s exact test. 

Calculating seroprevalence of SARS-CoV-2, the unweighted SARS-CoV-2 seroprevalence was the proportion of the individuals positive for anti-SARS-CoV-2 antibodies, whereas the weighted prevalence was adjusted for age and sex to the population of the study county based on the Kenya National Census of 2019 [23]. Seroprevalence was reported as the point estimates and 95% confidence intervals. The frequencies of the ratio of positive versus negative (P/N ratio) were plotted and a comparison between urban and rural populations’ immunoreactivity to SARS-CoV-2 was carried out. Any participant with a P/N ratio ≥ 1 was considered positive, and those with a P/N ratio ≥ 20 were classified as strongly immunoreactive. Statistical significance was set at *p* < 0.05 and the 95% confidence level and calculated as previously described [24]. 

For factors associated with vaccine uptake and refusal, separate multivariable Poisson regression for two outcome measures (vaccine uptake and vaccine refusal) were fitted to assess the independent associations with explanatory variables. In the univariable analysis, explanatory variables that were selected a priori were evaluated and the crude prevalence ratio and corresponding *p*-values with the outcome were determined. Explanatory variables that had a *p*-value < 0.2 were moved into the multivariable model. 

Multivariable regression was then applied to identify independent explanatory factors associated with the outcome variables (either vaccine uptake or refusal) and estimate the magnitude of the adjusted prevalence ratios (aPR) for the assessed factors. Model selection was conducted using the stepwise backward elimination method with the Akaike information criterion values used for model selection. The 95% confidence intervals (CIs) were computed for the aPR with statistical significance set at a *p*-value < 0.05. Model goodness of fit was assessed by the Hosmer–Lemeshow test. 

### 2.7. Ethical Considerations

This study was reviewed and approved by the Kenya Medical Research Institute Scientific and Ethical Review Committee (number SSC 4098) and the National Commission for Science, Technology, and Innovation (License number NACOSTI/P/21/13539). Additional ethical approval was provided by the University of Nairobi Institutional Review Board (Approval number P223/03/2022). Administrative approvals were provided by the Nairobi and Kakamega County health departments. All study participants provided written assents (for those aged 12–17 years) or consents (for those aged ≥ 18 years) before enrolment.

## 3. Results

### 3.1. Study Participants

We enrolled 1591 participants, 781 (49.1%) from the urban site with a median age of 29 years (IQR 20), and 810 (50.9%) from the rural site with a median age of 26 years (IQR 36). Overall, 29.9% (n = 476) of the participants were <18 years old, while 10.0% (n = 159) were ≥60 years, as shown in Table 1.

### 3.2. SARS-CoV-2 Seroprevalence

**Figure 2:**
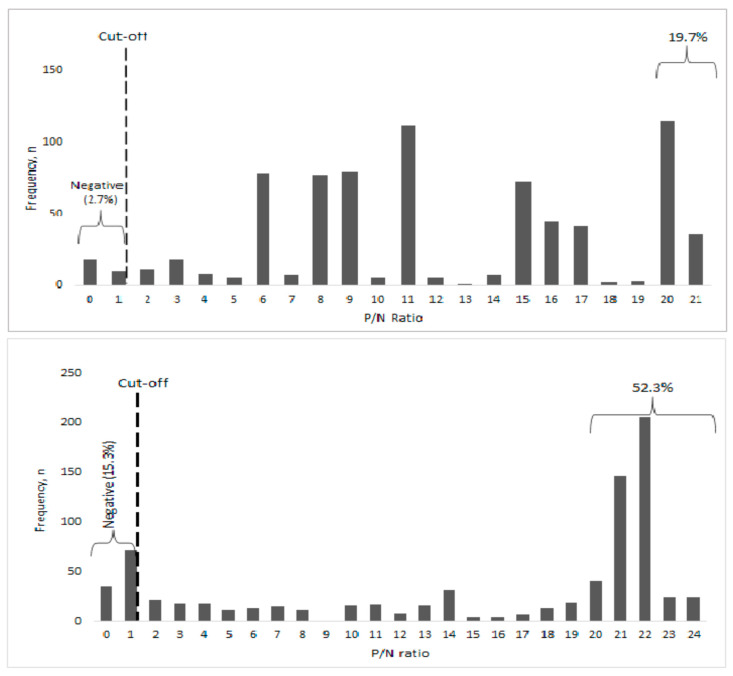
Frequency of the P/N ratio among urban (top panel) and rural (bottom panel) participants in Kenya, February 2022. Results were expressed as positive/negative ratios, with a value ≥ 1 considered positive. A significantly higher proportion of rural participants were more immunoreactive, suggesting recent infection or vaccination.

Serum samples from 1565 participants were tested for SARS-CoV-2 antibodies, 769 (49.1%) from urban and 796 (50.9%) from rural counties. The untested samples did not meet the quality control thresholds. Conclusive test results were obtained for 1537 (98.2%) participants, while the rest (1.8%) were indeterminate. The overall unweighted SARS-CoV-2 seroprevalence was 91.0% (95% CI, 89.4–92.3%), while the weighted prevalence was 90.2% (95% CI, 88.6–91.2%). In the urban county, the unweighted seroprevalence was 97.3% (95% CI, 95.8–98.3%), while the weighted prevalence was 96.7% (95% CI, 95.2–97.9%). In the rural county, the unweighted seroprevalence was 84.7% (95% CI, 82.0–87.2%), while the weighted prevalence was 83.6% (95% CI, 80.6–86.0%). The comparison of immunoreactivity (total IgM and IgG antibodies) against SARS-CoV-2 between participants from the two groups showed that 52.3% of seropositive rural participants were significantly more immunoreactive (P/N ratio ≥ 20, *p* < 0.001) when compared to <20% of the seropositive urban participants (Figure 2). In contrast, a larger proportion (15.3%) of rural participants were seronegative to the virus, when compared to 2.7% of the urban participants (*p* = <0.001). 

### 3.3. Vaccine Knowledge and Uptake

**Figure 3:**
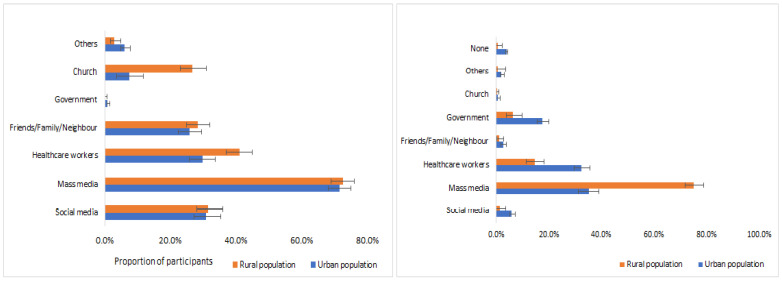
Sources of COVID-19 vaccine information (left panel) and the most trusted sources of COVID-19 vaccine information (right panel) among the urban and rural study participants.

Of the enrolled participants, 1254 (79.3%) who were ≥12 years old, including 670 (53.4%) from the urban and 584 (46.6%) from the rural counties, participated in the assessment of COVID-19 vaccine knowledge, attitude, and uptake. Almost all participants (97.5% urban, 97.6% rural) were aware of the availability of the COVID-19 vaccines. Among those aware, 70.0% of urban and 74.2% of rural participants knew that it protected those vaccinated, however, only 39.2% of urban and 40.4% of rural participants knew that community vaccination might also protect people who did not receive the vaccine (e.g., children). Mass media was the most common source of vaccine information (71.7% urban, 72.6% rural), and the most trusted (35.2% urban, 75.1% rural). Other less used and trusted sources of information included government workers, healthcare workers, and churches, as shown in Figure 3. Compared to urban participants, a higher proportion of rural participants reported their source of COVID-19 information as church or healthcare workers. A higher proportion of urban participants compared to rural participants trusted government or healthcare workers.

**Figure 4:**
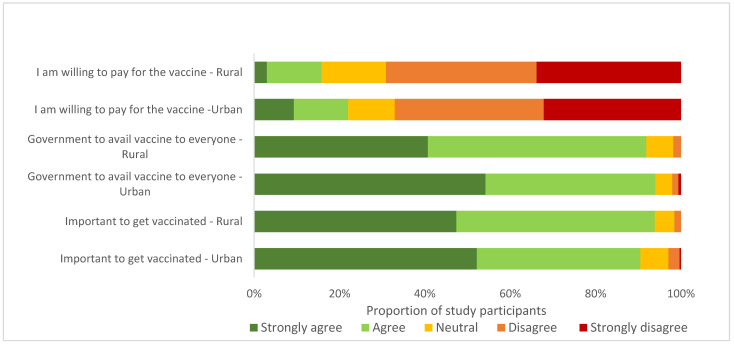
Participants’ attitudes towards the COVID-19 vaccine.

Over 90% of the participants (90.5% urban, 93.9% rural) agreed that vaccines were necessary, and that the government should make them available for everyone eligible (Figure 4).

Of 1115 enrolled participants that were ≥18 years old and therefore vaccine-eligible, 52.4% (n = 326) of urban and 53.1% (n = 262) of rural participants had received at least one dose of the vaccine, despite widespread availability and public education of the vaccine at least 3 months before the study was conducted. Among vaccinated participants, 40.7% had received AstraZeneca, 35.3% received Johnson and Johnson, 12.6% received Pfizer, and 11.4% received Moderna. Reasons given for uptake of the vaccine included perceived high-risk health status (26.7% urban, and 31.5% rural), belief in vaccine effectiveness (24.9% urban, 4.9% rural), and government directive on mandatory vaccination for certain groups (18.2% urban, 22.0% rural), as shown in Table 2.

The vaccination rate among those previously diagnosed with COVID-19 was 81.8%, and 53.6% among those with chronic medical conditions. Although age, occupation, previous COVID-19 diagnosis, vaccine knowledge and attitudes were significantly associated with vaccine uptake on univariable analysis (Table S2), only age, occupation, past COVID-19 diagnosis, and positive vaccine attitudes remained significant on multivariable analysis. Compared to the 18–30 years age group, urban participants aged > 30 years were up to 1.7 times more likely to have been vaccinated, while the rural participants aged 31–40 years were 1.6 times more likely to have been vaccinated (Table 3). Among the urban population, persons in formal employments (aPR 1.68, 95% CI: 1.06, 2.59) and those previously diagnosed with COVID-19 (aPR 1.54, 95% CI: 1.05, 2.20) were more likely to be vaccinated; whereas among the rural population, students were 2.5 times more likely to be vaccinated. 

### 3.4. Vaccine Refusal

Among the 1115 vaccine-eligible participants, 47.9% (298/622) of urban and 46.9% (231/493) of rural participants had not been vaccinated. Among the eligible participants, 19.6% (122/622) of urban and 15.6% (77/493) of rural participants indicated that they would never take the vaccine. Key reasons for vaccine refusal included concern about its safety, side effects, and a lack of information (Table 4). 

Although occupation, history of chronic breathing problems, source of COVID-19 vaccine information, vaccine knowledge and attitudes were significantly associated with vaccine refusal on univariable analysis (Table S3), only participants’ occupation and COVID-19 vaccine attitudes were significantly associated with vaccine refusal on multivariable analysis. Those employed in informal sectors were less likely to refuse the vaccine (aPR 0.20, 95% CI: 0.05, 0.88). Urban participants who disagreed or strongly disagreed with the importance of vaccines were more likely to refuse the vaccine (aPR 2.57, 95% CI: 1.32, 4.58). Age, level of education, and history of chronic medical conditions were not significantly associated with vaccine refusal (Table 5).

## 4. Discussion

Two years after the first case of COVID-19 was confirmed and six months after the widespread availability of vaccines, we found high levels (84–97%) of population immunity against the SARS-CoV-2 virus among both rural and urban populations of Kenya. This herd immunity was observed despite low levels (<55%) of vaccine uptake and high levels (16–20%) of vaccine refusal among the two populations. Widespread SARS-CoV-2 transmission across all communities in the country is the most plausible explanation for this herd immunity, an argument supported by the findings of significantly higher seroprevalence in urban (97.8%) than rural (85.9%) populations. Throughout the pandemic, Nairobi City, densely populated (>4 million people) with >65% of residents living in informal settlements, served as a hotspot of the pandemic [25]. In contrast, immunity among residents of the rural Kakamega County was lower and included a >14% naive population, perhaps reflecting lower community transmission of the virus in such rural areas [26]. When we compared the spectrum of immunity against the virus between the two populations, we found >50% of the seropositive rural population strongly immunoreactive when compared to <20% in the rural population, suggesting recent infection, likely linked to the Omicron variant that dominated the fifth wave ongoing at the time of the study. In contrast, the level of immunity in the urban population was evenly spread, reflecting recurrent infections, including breakthrough infections and vaccination.

The population immunity to SAR-CoV-2 reported in this study, which was higher than in studies conducted in Kenya during the early phases of the pandemic [25], reflected a continuous high-transmission rate of the virus despite global introduction of vaccines in March 2021 [7]. Scientists projected that at least 70% of the population needed to be immunized against SARS-CoV-2 to achieve the herd immunity needed to break virus transmission [27,28]. Considering the morbidity profile of SARS-CoV-2, the global health community led by the World Health Organization (WHO) contended that herd immunity needed to be achieved through vaccination rather than natural infection [29]. However, the inequities in COVID-19 vaccine availability to low- and medium-income countries experienced in the early phase likely resulted in herd immunity in many countries being driven by natural infections rather than vaccination [30]. While our findings clearly show that there likely is only a small population of SARS-CoV-2-naive individuals, COVID-19 vaccination programs must continue because of studies showing rapid immunity decay and high prevalence of breakthrough infections associated with the continued emergence of new virus variants [31,32].

The COVID-19 vaccination efforts faced considerable hesitancy, with <50% of the population having started receiving doses by February 2022, almost a year after the introduction of vaccines [21]. Our study found participants who had previously been diagnosed with COVID-19 and older populations more likely to be vaccinated, suggesting that people that felt more susceptibility to the disease were more likely to accept the intervention [33]. However, some studies have shown no association between past infection with COVID-19 and vaccine uptake [34]. Our study found participants engaged in formal employments more likely to be vaccinated, perhaps associated with vaccine mandates imposed by the government and employers [35]. As shown in other studies, participants who did not think vaccines were important in controlling the pandemic were >2 times more likely to be vaccine-hesitant [36].

Our study found up to 20% of the eligible population refusing to take the COVID-19 vaccine. While this proportion may not be large enough to prevent the achievement of herd immunity, it may create pockets of SARS-CoV-2 transmission and associated morbidities and mortalities in the future. The COVID-19 vaccine refusal levels reported in other African countries ranged from 6% to 61%; however, these studies often failed to segregate hesitancy from refusal [37]. Among the reasons given for vaccine refusal in our study included concerns about safety (>75%), inadequate information (26%), and concerns about effectiveness (9%). Interestingly, the commonly cited reasons for vaccine refusal such as religious or cultural beliefs were cited by less than 2% of the participants in our study [10,38]. Participants cited mass media as the most common and trusted source of COVID-19 vaccine information, contrary to studies in the early phase of the pandemic that found mass media less trusted than healthcare workers, the government, and religious leaders [39,40]. Mass media may have gained traction over time by focusing on myths, vaccine safety concerns, and side effects, which were identified early as common reasons for vaccine hesitancy and refusal [41,42]. 

The study had some limitations. First, we did not verify the vaccination status of all participants who reported being vaccinated, possibly indicating that the vaccine uptake may have been lower that reported. Second, our serological test did not differentiate between infection- and vaccination-induced SARS-CoV-2 antibodies, which would have more clearly defined the levels of population immunity induced by the two pathways. However, given the introduction of multiple types of vaccines, the commonly suggested comparative ELISA between SARS-CoV-2 anti-spike protein and anti-nucleoprotein would not have provided a conclusive answer. 

## 5. Conclusions

Despite considerable COVID-19 vaccine hesitancy and refusal, Kenya and likely other African countries had achieved herd immunity by early 2022, with natural infections contributing almost 50% of this immunity. However, due to the rapidly waning SARS-CoV-2 immunity and emergence of immune-evading virus variants, vaccine campaigns with upgraded vaccines should be sustained globally. There is considerable vaccine hesitancy and refusal; however, its impact on controlling the pandemic appears minimal, with almost all participants having attained antibodies against SARS-CoV-2. 

## Figures and Tables

**Table 1:** Sociodemographic characteristics of all study participants.

	Urban Participants, N = 781		Rural Participants, N = 810	
Characteristic	n (%)	95% CI (%)	n (%)	95% CI (%)
Sex				
Female	497 (63.6)	60.0, 67.0	469 (57.9)	54.0, 61.0
Male	284 (36.4)	33.0, 40.0	341 (42.1)	39.0, 46.0
Age group (years)				
≤9	94 (12.0)	9.9, 15.0	188 (23.2)	20.0, 26.0
10–19	94 (12.0)	9.9, 15.0	154 (19.0)	16.0, 22.0
20–29	215 (27.5)	24.0, 31.0	96 (11.9)	9.7, 14.0
30–39	174 (22.3)	19.0, 25.0	108 (13.3)	11.0, 16.0
40–49	93 (11.9)	9.8, 14.0	84 (10.4)	8.4, 13.0
50–59	65 (8.3)	6.5, 11.0	67 (8.3)	6.5, 10.0
60+	46 (5.9)	4.4, 7.8	113 (14.0)	12.0, 17.0
Level of education				
Primary	265 (34.1)	31.0, 38.0	198 (24.5)	22.0, 28.0
Secondary	215 (27.7)	25.0, 31.0	116 (14.3)	12.0, 17.0
Post-secondary	168 (21.6)	19.0, 25.0	35 (4.3)	3.1, 6.0
Child	102 (13.1)	11.0, 16.0	223 (27.6)	25.0, 31.0
No formal education	27 (3.5)	2.3, 5.1	237 (29.3)	26.0, 33.0
Missing	4 (0.5)	0.1, 1.3	1 (0.1)	0.0, 0.7
Occupation				
Child (<18 years)	159 (20.4)	18.0, 23.0	317 (39.1)	36.0, 43.0
Student	44 (5.6)	4.2, 7.6	24 (3.0)	1.9, 4.4
Informal employment	187 (23.9)	21.0, 27.0	46 (5.7)	4.2, 7.6
Formal employment	35 (4.5)	3.2, 6.2	20 (2.5)	1.6, 3.9
Self-employed	161 (20.6)	18.0, 24.0	267 (33.0)	30.0, 36.0
Healthcare worker	10 (1.3)	0.65, 2.4	2 (0.2)	0.04, 1.0
Unemployed	181 (23.2)	20.0, 26.0	126 (15.6)	13.0, 18.0
Other	4 (0.5)	0.16, 1.4	8 (1.0)	0.6, 2.0
Reported chronic illness *				
No	668 (85.5)	83.0, 88.0	721 (89.0)	87.0, 91.0
Yes	113 (14.5)	12.0, 17.0	89 (11.0)	9.0, 13.0
Ever diagnosed with COVID-19				
No	738 (94.5)	93.0, 96.0	809 (99.9)	99.0, 100
Yes	43 (5.5)	4.1, 7.4	1 (0.1)	0.01, 0.80

* Chronic breathing problems, heart disease, hypertension, stroke, diabetes, cancer, liver disease, tuberculosis, kidney disease.

**Table 2:** Reasons for COVID-19 vaccine uptake among vaccinated urban and rural study participants.

	Urban Participants, N = 326		Rural Participants, N = 262	
Reasons for COVID-19 Vaccine Uptake	n (%)	95% CI (%)	n (%)	95% CI (%)
Perceived high-risk health status	108 (26.7)	23.0, 31.0	106 (31.5)	27.0, 37.0
Vaccine effectiveness	98 (24.9)	21.0, 30.0	12 (4.9)	2.7, 8.7
GOK directive	66 (18.2)	14.0, 23.0	65 (22.0)	17.0, 27.0
Number of COVID-19 deaths	53 (15.2)	12.0, 20.0	60 (20.6)	16.0, 26.0
Employer requirement	45 (13.2)	9.9, 17.0	8 (3.3)	1.6, 6.7
Number of COVID-19 cases	43 (12.7)	9.4, 17.0	97 (29.6)	25.0, 35.0
Suggestions from healthcare workers	24 (7.5)	5.0, 11.0	35 (13.2)	9.5, 18.0
Suggestions from family/friends/neighbor	18 (5.7)	3.5, 9.1	4 (1.7)	0.6, 4.6
Advanced age	11 (3.6)	1.9, 6.5	19 (7.6)	4.8, 12.0
Free vaccine	11 (3.6)	1.9, 6.5	11 (4.5)	2.4, 8.2
Others *	12 (3.9)	2.1, 6.9	4 (1.7)	0.6, 4.6

* Country of vaccine origin, number of doses, type of vaccine, for travel purposes.

**Table 3:** Factors associated with COVID-19 vaccine uptake among the urban and rural study participants.

	Urban Participants			Rural Participants		
Characteristic	aPR ^1^	95% CI ^1^	*p*-Value	aPR ^1^	95% CI ^1^	*p*-Value
Age group						
18–30	Ref	Ref		Ref	Ref	
31–40	1.42	1.05, 1.91	0.021	1.35	0.88, 2.12	0.2
41–50	1.68	1.18, 2.37	0.004	1.58	1.01, 2.51	0.046
51–60	1.49	1.00, 2.18	0.042	1.42	0.88, 2.31	0.15
61+	1.60	1.00, 2.47	0.041	1.38	0.89, 2.17	0.2
Main occupation						
Unemployed	Ref	Ref		Ref	Ref	
Self-employment	1.10	0.80, 1.51	0.6	1.40	1.00, 2.01	0.060
Formal employment	1.68	1.06, 2.59	0.023	1.60	0.86, 2.86	0.12
Student	1.37	0.81, 2.25	0.2	2.50	1.32, 4.60	0.004
Informal employment	1.16	0.85, 1.59	0.4	1.17	0.67, 1.95	0.6
Healthcare worker	1.79	0.86, 3.35	0.091	1.04	0.06, 4.90	>0.9
Diagnosed with COVID-19				NA *		
No	Ref	Ref				
Yes	1.54	1.05, 2.20	0.021			
COVID-19 Vaccine attitudes Vaccines are important				NA *		
Strongly Agree/Agree	Ref	Ref				
Neutral	0.62	0.33, 1.05	0.10			
Strongly Disagree/Disagree	0.23	0.04, 0.71	0.037			
Vaccine protects against infection				NA *		
No/Don’t know	Ref	Ref				
Yes	1.12	0.87, 1.44	0.4			
Source of COVID-19 information Social media				NA *		
No	Ref	Ref				
Yes	1.26	1.00, 1.58	0.052			
COVID-19 Knowledge Vaccine protects the unvaccinated	NA *					
Yes				Ref	Ref	
No/Don’t know				0.88	0.68, 1.13	0.3
Children can be vaccinated	NA *					
No/Don’t know				Ref	Ref	
Yes				1.22	0.95, 1.57	0.12
Vaccine has no side effects	NA *					
No/Don’t know				Ref	Ref	
Yes				1.14	0.87, 1.48	0.4
COVID-19 Vaccine attitudes I trust the COVID-19 vaccine information from the media	NA *					
Neutral				Ref	Ref	
Strongly Agree/Agree				1.43	0.81, 2.80	0.3
Strongly Disagree/Disagree				1.47	0.58, 3.61	0.4
Source of COVID-19 vaccine information Mass Media	NA *					
No				Ref	Ref	
Yes				1.17	0.86, 1.61	0.3

aPR ^1^ = adjusted prevalence ratio, CI ^1^ = confidence interval, NA * = not assessed.

**Table 4:** Reasons for vaccine refusal among those not vaccinated.

	Urban Participants, N = 122		Rural Participants, N = 77	
Reasons for COVID-19 Vaccine Refusal *	n (%)	95% CI	n (%)	95% CI
Concerns about side effects	55 (45.1%)	36%, 54%	41 (53.2%)	42%, 65%
Concerns about vaccine safety	36 (29.5%)	22%, 39%	22 (28.6%)	19%, 40%
Lack of vaccine information	32 (26.2%)	19%, 35%	19 (24.7%)	16%, 36%
Concerns about vaccine effectiveness	11 (9.0%)	4.8%, 16%	2 (2.6%)	0.45%, 9.9%
Vaccine can cause COVID-19	3 (2.5%)	0.64%, 7.6%	2 (2.6%)	0.45%, 9.9%
Religious reasons	1 (0.8%)	0.04%, 5.2%	2 (2.6%)	0.45%, 9.9%
Cultural reasons	1 (0.8%)	0.04%, 5.2%	2 (2.6%)	0.45%, 9.9%
Others **	30 (24.6%)	17%, 33%	9 (11.7%)	5.8%, 22%

* Multiple responses per participant allowed. ** Not interested, pregnant, underlying medical conditions, COVID-19 cases have gone down.

**Table 5:** Factors associated with COVID-19 vaccine refusal among study participants.

	Urban Participants, N = 122			Rural Participants, N = 77		
Characteristic	aPR ^1^	95% CI ^1^	*p*-Value	aPR ^1^	95% CI ^1^	*p*-Value
Age group						
18–30	Ref	Ref		Ref	Ref	
31–40	1.24	0.80, 1.89	0.3	1.65	0.80, 3.37	0.2
41–50	0.59	0.20, 1.34	0.3	1.38	0.54, 3.52	0.5
51–60	1.21	0.61, 2.19	0.6	1.20	0.50, 2.90	0.7
61+	0.58	0.14, 1.60	0.4	1.20	0.57, 2.51	0.6
Level of education		NA *				
No formal education				Ref	Ref	
Primary				0.66	0.36, 1.21	0.2
Secondary				0.52	0.26, 1.04	0.066
Post-secondary				1.02	0.37, 2.77	>0.9
Main Occupation		NA *				
Unemployed				Ref	Ref	
Self Employed				0.73	0.44, 1.22	0.2
Employed				0.76	0.15, 3.78	0.7
Student				1.74	0.55, 5.53	0.3
Informal Employment				0.20	0.05, 0.88	0.033
Healthcare worker				0.00	0.00, Inf	>0.9
Willing to pay for vaccine privately		NA *				
Neutral				Ref	Ref	
Strongly Disagree/Disagree				1.46	0.69, 3.11	0.3
Strongly Agree/Agree				0.55	0.14, 2.16	0.4
Trust in vaccine information from the media		NA *				
Neutral				Ref	Ref	
Strongly Agree/Agree				0.49	0.25, 0.94	0.032
Strongly Disagree/Disagree				0.72	0.18, 2.79	0.6
Vaccines are important						
Strongly Agree/Agree	Ref	Ref				
Neutral	2.09	1.21, 3.43	0.005			
Strongly Disagree/Disagree	2.57	1.32, 4.58	0.003			
History of chronic breathing problems						
No	Ref	Ref				
Yes	1.90	0.98, 3.38	0.041			
History of hypertension						
No	Ref	Ref				
Yes	0.54	0.19, 1.23	0.2			
Unknown	1.39	0.08, 6.31	0.7			

aPR ^1^ = adjusted prevalence ratio, CI ^1^ = confidence interval, NA * = not assessed.

## Data Availability

The data presented in this study are available upon request from the corresponding author.

## Supplementary Materials

The following are available online at https://www.mdpi.com/article/10.3390/vaccines11010068/s1. Table S1: Study questionnaire; Table S2: Factors associated with COVID-19 vaccine uptake in the univariable analysis; Table S3: Factors associated with COVID-19 vaccine refusal in the univariable analysis.
