# Supplementary material for: Near-Complete SARS-CoV-2 Seroprevalence among Rural and Urban Kenyans despite Significant Vaccine Hesitancy and Refusal
Source: Vaccines (Basel). 2022 Dec 28;11(1):68. doi: 10.3390/vaccines11010068 (PMC9862465; doi:10.3390/vaccines11010068)
Supplement: Supplementary file 1 [file vaccines-11-00068-s001.zip › vaccines-2028304-supplementary.pdf]

Table S1: Study Questionnaire

**A1. Household ID (From list):**	**Household Head Contacts:**	**A2. Date of interview: (dd/mm/yy)**
**A3. Enumerator initials:**	**A4. County:**<br>**A5. Sub-county:**	
**A6. HH geographic coordinates**		

SECTION B. HOUSEHOLD DEMOGRAPHICS ALL HOUSEHOLD MEMBERS CURRENTLY LIVING WITHIN LAST WEEK

**B1. Household Member No.**	**B2. Position in the Household** (in relation to the household head)	**B3. Age (years)**	**B4. Age in months (If less than 1 year)**	**B5. Sex**	**B6. Highest level of formal education completed**	**B7. Primary occupation (Pick one)**
	1=Household Head<br>2=Spouse of Household Head<br>3=Son/Daughter<br>4=Sibling<br>5=Father/Mother<br>6=Nephew/niece<br>7=Grand (son/daughter)<br>8=Herdsman/woman<br>9= House help<br>10=Other (Specify above)	(Years)	Months	1=Male<br>2=Female	0= Child<br>1=No formal education<br>2 = Primary<br>3 = Secondary<br>4 = Postsecondary<br>5 = Other	1. Student<br>2. Farmer<br>3. Business Owner<br>4. Civil servant<br>5. Health care worker<br>6. Skilled Worker<br>7. Unskilled laborer<br>8. Not disclosed

B8. Relation of the person interviewed to the household head:
☐Household head ☐Spouse ☐Son/Daughter ☐Other Relative
☐Caretaker

INDIVIDUAL SECTION

SECTION C: RESPIRATORY ILLNESS AND MEDICAL HISTORY

C1. In the last 14 days have you had a respiratory illness with (cough or sore throat or running nose)? ☐Yes ☐ No ☐ Unknown

C2 Do you currently having any of the following symptoms (cough or sore throat or running nose)?

☐Yes ☐ No

IF YES TO ANY OF THE SYMPTOMS, AN NP/OP SWABS WILL BE COLLECTED FROM THE PARTICIPANT

C3 Have you had any of these symptoms in the last 14 days (check all that apply)

Symptom	No. of Days since onset
☐Fever (or history of fever)	________________
☐Cough	________________ ☐
Sore throat	________________
☐Running nose	________________
☐Chills	________________
☐Fatigue	________________
☐Muscle ache (myalgia)	________________
☐Shortness of breath (dyspnea)	________________
☐Wheezing	________________
☐Chest pain	________________
☐Headache	________________
☐Nausea/vomiting	________________
☐other (specify)	________________

C4. In the last 14 days have you been unable to perform daily chores because of a respiratory illness?

☐Yes ☐ No ☐ Can't remember

C5. If yes, estimated # days______________

C6. In the last 14 days has anyone in your household been ill with a respiratory illness (running nose and cough or sore throat)? ☐Yes ☐ No ☐ Unknown

C7. If yes, number of household members with respiratory illness in the last 14 days

☐1 ☐ 2 ☐ 3 ☐ 4 ☐ ≥5

C8. In the last 14 days has anyone in your household been hospitalized due to respiratory illness?

☐Yes ☐ No

C9. In the last 14 days has anyone at work been ill with a respiratory illness (fever and cough or sore throat)? ☐Yes ☐ No ☐ Unknown ☐Don't work away from home

C10. In the last 14 days has anyone at work been hospitalized due to respiratory illness?
☐Yes ☐ No ☐ Unknown

C11. Since the beginning of this year, have you had a respiratory illness?
☐Yes ☐ No ☐ Unknown

Medical and smoking history

C12. Do you have a medical history of any chronic breathing problems, such as asthma, coughing?
☐Yes ☐ No ☐ Unknown

C13. Do you have a medical history of heart disease, hypertension, or stroke?
☐Yes ☐ No ☐ Unknown

C14. Do you have a medical history of any other chronic medical problems, such as diabetes, cancer, liver disease, kidney disease, tuberculosis?
☐Yes ☐ No ☐ Unknown

C 15. Have you ever been diagnosed with COVID-19 since the pandemic started?
☐ Yes ☐ No

C 16. Has any member of your household ever been diagnosed with COVID-19?
☐ Yes ☐ No

C 17. Has any one of your relatives ever been diagnosed with COVID-?
☐Yes ☐No ☐Don't Know

C 18. Has any one of your friends or neighbors been diagnosed with COVID-?
☐Yes ☐No ☐Don't know

SECTION D: COVID-19 KNOWLEDGE, ATTITUDES AND PRACTICES

D1. Do you think that people who show no symptoms of being sick, such as coughing or sneezing, can spread a virus / contagious disease? ☐Yes ☐ No

D2. Do you think it may be possible that a person can get a virus / contagious by touching a surface or object that has the virus on it? ☐Yes ☐ No

D3. Do you have a surgical or a homemade cloth mask that you use to cover your mouth or nose when you leave your house? ☐Yes ☐ No

D4. In the past 7 days, have you worn it every time you left your house? ☐Yes ☐ No

D5. For how many days, have you stayed at home all day, without going out at all and without receiving any visits? _____

D6. Why did you stay at home all day? **[DO NOT READ OUT OPTIONS - SELECT ALL THAT APPLY]**

☐ There was a mandatory lockdown
☐ It was recommended by the government / local authority
☐ It was recommended by health experts
☐ I was asked to stay at home by a family member, friend, or other acquaintance
☐ I was worried about getting infected
☐ I was worried about spreading the virus
☐ There was no work
☐ Worked from home
☐ None of the above

D7. In the past 7 days, did you attend social gatherings (e.g., visit family and friends, drink tea at a stall, etc.) ☐ Yes ☐ No

D8. In the past 7 days, did you cover your mouth and nose with your bent elbow when you coughed or sneezed? ☐ Yes ☐ No

D9. In the past 7 days, did you leave your home to do some work? ☐ Yes ☐ No

SECTION E: COVID-19 VACCINE KNOWLEDGE AND ATTITUDES

COVID-19 Vaccine knowledge

E1. Are you aware of the COVID-19 vaccine? Yes No

E2. If yes to E1,

E2a. What was the source of your COVID-19 vaccine related information?

☐ social media ☐ Mass media ☐ Health care workers ☐ Friends/family/neighbors

E2b. Who do you trust the most for information about vaccines?

☐ Media (TV, Radio, Newspaper)
☐ Internet
☐ Social media (Facebook, Twitter, WhatsApp, .etc.)
☐ Health care providers: Physicians, pharmacists, etc.
☐ Family-members
☐ Government (JFDA)
☐ The pharmaceutical company reports
☐ Scientific articles
☐ I do not trust any source

E2c. How is the vaccine administered?

☐ Via injection ☐ Orally ☐ Other routes

E2d. Do you know how many does of the vaccine does one require? ☐ Yes ☐ No ☐ Don't know

For E2e-E2h, tick appropriate responses.

E2e. COVID_19 vaccine protects the receiver from getting infection.

☐ Yes ☐ No ☐ Don't know

E2f. COVID-19 vaccine may protect other people who do not receive the vaccine.

☐ Yes ☐ No ☐ Don't know

E2g. Everyone including children can receive the vaccine. ☐ Yes ☐ No ☐ Don't know

E2h. COVID-19 vaccines do not have side effects. ☐ yes ☐ No ☐ Don't know

COVID-19 Vaccine Attitudes

E3._Kindly indicate the extent to which you agree or disagree with each of the following statements.

Statement	Strongly disagree	Disagree	Neutral	Strongly agree	Agree
It is important to get a vaccine to protect the people from COVID-19					
The government should make the vaccine available for all citizens.					
I am willing to pay for the COVID-19 vaccine privately					
I trust in the information provided by media about the COVID-19 vaccine.					

COVID-19 vaccine acceptance

E3. Have you been vaccinated against COVID-19? ☐ Yes ☐ No

E4. If yes,

How many doses of the vaccine have you received? ☐ 1 ☐ 2

E5 Which vaccine did you receive?

☐ AstraZeneca

☐ Pfizer

☐ Moderna

☐ Johnson and Johnson

☐ Sinopharm

☐ Don't know

E6. If 1 dose, do you plan on receiving your second dose? ☐ Yes ☐ No

Proof of vaccination available. ☐ Text message from MOH ☐ Vaccination certificate
☐ Others ☐ Proof not available

E7. What factors influence your decision to take the vaccine?
☐ Number of confirmed cases ☐ Suggestions from doctors/Ministry of Health
☐ Number of COVID-19 related deaths ☐ Suggestions from family/friends/neighbors
☐ Health status ☐ Type of vaccine
☐ Age ☐ Number of doses
☐ Vaccine effectiveness ☐ Country that produces the vaccine
☐ Vaccine available for free ☐ Others(specify)

If no to Qs E3, would you receive the COVID-19 vaccine if offered to you? ☐ Yes ☐ No
If no, why would you not receive the vaccine?
☐ lack of information regarding the vaccine ☐ vaccine can cause COVID-19
☐ side effects ☐ Religious reasons
☐ safety concerns ☐ Cultural reasons
☐ effectiveness concerns ☐ Others(specify)

SECTION F: LABORATORY INFORMATION

F1. Was a specimen collected? 1=Yes 2=No
If no, reason?
…….
F3. Date(s) of specimen collection: …………………………………………….
F4. Specimen type: NP Swab OP Swab Serum

Table S2: Factors associated with COVID-19 vaccine uptake on univariable analysis

	Nairobi (Urban) n=326				Kakamega (Rural) n=262			
Characteristic	Vaccinated	PRR[1]	95% CI[1]	p-value	Vaccinated	PRR[1]	95% CI[1]	p-value
Sex								
Female	214	—	—		166	—	—	
Male	112	1.10	0.87, 1.37	0.43	96	1.10	0.85, 1.41	0.46
Age group								
18-30	103	—	—		51	—	—	
31-40	101	1.41	1.07, 1.85	0.015	60	1.35	0.93, 1.96	0.12
41-50	56	1.66	1.19, 2.29	0.002	56	1.65	1.13, 2.41	0.010
51-60	39	1.48	1.01, 2.12	0.037	39	1.46	0.96, 2.21	0.075

	Nairobi (Urban) n=326				Kakamega (Rural) n=262			
Characteristic	Vaccinated	PRR[1]	95% CI[1]	p-value	Vaccinated	PRR[1]	95% CI[1]	p-value
61+	27	1.55	0.99, 2.33	0.043	56	1.29	0.88, 1.89	0.18
Age in years	326	1.01	1.00, 1.02	0.002	262	1.00	1.00, 1.01	0.43
Level of education								
No formal education	11	—	—		72	—	—	
Primary	95	1.03	0.58, 2.05	0.91	102	1.28	0.95, 1.74	0.11
Secondary	110	1.25	0.70, 2.46	0.48	66	1.28	0.92, 1.79	0.15
Post-secondary	109	1.54	0.87, 3.04	0.17	21	1.35	0.81, 2.15	0.23
Main Occupation								
Unemployed	78	—	—		45	—	—	
Self Employed	89	1.28	0.95, 1.74	0.11	160	1.69	1.23, 2.38	0.002
Employed	28	1.80	1.15, 2.74	0.007	17	2.18	1.21, 3.74	0.006
Student	21	1.11	0.67, 1.76	0.68	17	2.00	1.11, 3.42	0.015
Informal Employment	99	1.23	0.91, 1.66	0.17	21	1.29	0.75, 2.13	0.34
Health care worker	10	2.32	1.13, 4.27	0.012	1	1.41	0.08, 6.45	0.73
History of chronic illness								
Any chronic illness								
No	267	—	—		219	—	—	
Yes	59	1.08	0.80, 1.41	0.61	43	0.96	0.68, 1.31	0.79
Chronic breathing problems								
No	312	—	—		244	—	—	
Yes	14	0.78	0.43, 1.27	0.35	2	0.76	0.13, 2.36	0.70
Unknown					16	1.17	0.67, 1.87	0.55
Hypertension								
No	284	—	—		230	—	—	
Yes	41	1.25	0.89, 1.72	0.18	30	0.86	0.57, 1.23	0.43
Unknown	1	0.65	0.04, 2.88	0.67	2	0.46	0.08, 1.43	0.27
Diagnosed with COVID-19								
No	291	—	—		261	—	—	
Yes	35	1.66	1.15, 2.32	0.005	1	1.89	0.11, 8.35	0.53
Source of COVID-19 information								

	Nairobi (Urban) n=326				Kakamega (Rural) n=262			
Characteristic	Vaccinated	PRR[1]	95% CI[1]	p-value	Vaccinated	PRR[1]	95% CI[1]	p-value
Social media								
No	207	—	—		180	—	—	
Yes	118	1.28	1.02, 1.61	0.031	82	0.96	0.73, 1.24	0.75
Mass media								
No	102	—	—		60	—	—	
Yes	223	0.87	0.69, 1.10	0.24	202	1.15	0.87, 1.54	0.35
Health care workers								
No	218	—	—		147	—	—	
Yes	107	1.11	0.87, 1.39	0.40	115	1.05	0.82, 1.33	0.71
Family/friends/neighbors								
No	249	—	—		190	—	—	
Yes	76	0.91	0.70, 1.17	0.46	72	0.89	0.68, 1.17	0.42
Church								
No	301	—	—		191	—	—	
Yes	24	1.02	0.66, 1.51	0.92	71	0.93	0.71, 1.22	0.61
Others								
No	305	—	—		255	—	—	
Yes	20	1.11	0.68, 1.69	0.66	7	1.18	0.50, 2.31	0.67
Trusted source of COVID-19 information								
Mass media	105	—	—	—	191	—	—	
Social media	18	1.02	0.60, 1.64	0.94	3	1.14	0.28, 2.98	0.83
Health Care providers	115	1.13	0.87, 1.47	0.37	44	1.14	0.81, 1.57	0.43
Family-members	4	0.53	0.16, 1.26	0.21	1	0.38	0.02, 1.68	0.33
I do not trust any source	5	0.40	0.14, 0.87	0.043	2	1.90	0.31, 5.91	0.37
Government	66	1.22	0.89, 1.66	0.20	18	1.07	0.63, 1.68	0.80
Others	8	1.32	0.59, 2.54	0.45	1	0.63	0.04, 2.80	0.65
COVID-19 vaccine knowledge								
Vaccine protects against infection								
No/Don’t know	87	—	—		57	—	—	
Yes	236	1.16	0.91, 1.49	0.24	205	1.23	0.92, 1.66	0.17
Vaccine protects the unvaccinated								

	Nairobi (Urban) n=326				Kakamega (Rural) n=262			
Characteristic	Vaccinated	PRR[1]	95% CI[1]	p-value	Vaccinated	PRR[1]	95% CI[1]	p-value
Yes	126	—	—		119	—	—	
No/ Don't know	199	1.05	0.84, 1.32	0.66	143	0.82	0.65, 1.05	0.12
Children can be vaccinated								
No/ Don't know	201	—	—		128	—	—	
Yes	124	1.00	0.80, 1.25	>0.99	134	1.31	1.03, 1.67	0.029
Vaccine has no side effects								
No/ Don't know	236	—	—		168	—	—	
Yes	89	1.04	0.81, 1.32	0.76	94	1.17	0.90, 1.50	0.23
COVID-19 vaccine attitudes								
It's important to get a COVID-19 vaccine								
Strongly Agree/Agree	309	—	—		251	—	—	
Neutral	13	0.60	0.32, 0.99	0.067	7	0.55	0.23, 1.08	0.12
Strongly Disagree/Disagree	3	0.32	0.08, 0.82	0.047	4	0.90	0.28, 2.12	0.84
Government should avail vaccines to everyone								
Strongly Agree/Agree	316	—	—		249	—	—	
Strongly Disagree/Disagree	2	0.30	0.05, 0.94	0.092	2	0.45	0.07, 1.39	0.26
Neutral	7	0.58	0.25, 1.13	0.15	11	0.61	0.31, 1.07	0.11
I am willing to pay for the vaccine								
Neutral	35	—	—		37	—	—	
Strongly Disagree/Disagree	223	0.96	0.68, 1.40	0.84	174	0.90	0.64, 1.31	0.58
Strongly Agree/Agree	67	0.87	0.58, 1.33	0.51	51	1.21	0.80, 1.86	0.37
I trust the COVID-19 vaccine information from the media								
Neutral	51	—	—		11	—	—	
Strongly Agree/Agree	253	1.04	0.78, 1.43	0.78	241	1.77	1.02, 3.45	0.064
Strongly Disagree/Disagree	21	0.71	0.42, 1.16	0.18	10	1.99	0.83, 4.72	0.12

PRR[1] = Prevalence Rate Ratio, CI[1] = Confidence Interval

Table S3: factors associated with COVID-19 vaccine refusal on univariable analysis

	Urban n=122				Rural n=77			
Characteristic	Refusal	PRR[1]	95% CI[1]	p-value	Refusal	PRR[1]	95% CI[1]	p-value
sex								
Female	83	—	—		56	—	—	
Male	39	1.09	0.73, 1.58	0.67	21	0.80	0.47, 1.29	0.37
Age group								
18-30	61	—	—		21	—	—	
31-40	38	1.23	0.81, 1.83	0.32	19	1.36	0.72, 2.53	0.33
41-50	7	0.65	0.27, 1.32	0.27	7	0.89	0.35, 2.00	0.80
51-60	12	1.15	0.59, 2.06	0.66	9	1.19	0.52, 2.52	0.66

	Urban n=122				Rural n=77			
Characteristic	Refusal	PRR[1]	95% CI[1]	p-value	Refusal	PRR[1]	95% CI[1]	p-value
61+	4	0.62	0.19, 1.51	0.36	21	1.47	0.80, 2.71	0.21
Age in years	122	1.00	0.98, 1.01	0.53	77	1.01	1.00, 1.02	0.15
Level of education								
No formal education	6	—	—		38	—	—	
Primary	46	0.94	0.44, 2.46	0.89	21	0.65	0.37, 1.09	0.11
Secondary	36	0.91	0.41, 2.40	0.83	13	0.62	0.32, 1.13	0.13
Post-secondary	33	1.40	0.63, 3.71	0.45	5	0.85	0.29, 1.96	0.72
Main Occupation								
Unemployed	39	—	—		32	—	—	
Self Employed	32	1.14	0.71, 1.82	0.58	35	0.84	0.52, 1.35	0.47
Employed	4	1.32	0.40, 3.28	0.60	2	1.03	0.25, 4.28	0.97
Student	15	1.72	0.92, 3.06	0.074	4	1.46	0.52, 4.14	0.47
Informal Employment	31	0.93	0.58, 1.49	0.76	2	0.20	0.05, 0.86	**0.030**
Health care worker	0	0	0	0	0	0.00	0.00, Inf	>0.99
History of chronic illness								
Any chronic illness								
No	103	—	—		59	—	—	
Yes	19	0.96	0.57, 1.53	0.87	18	1.41	0.81, 2.35	0.20
Chronic breathing problems								
No	110	—	—		69	—	—	
Yes	12	1.52	0.79, 2.64	0.17	7	2.21	0.92, 4.48	0.045
Unknown					1	1.05	0.06, 4.75	0.96
Hypertension								
No	116	—	—		60	—	—	
Yes	5	0.49	0.17, 1.08	0.12	14	1.31	0.70, 2.27	0.36
Unknown	1	1.17	0.07, 5.23	0.87	3	1.59	0.39, 4.29	0.43
Diagnosed with COVID-19								
No	117	—	—		0	NA		
Yes	5	1.55	0.55, 3.41	0.34	0	NA		
Source of COVID-19 information								
Social media								
No	84	—	—		48	—	—	

	Urban n=122				Rural n=77			
Characteristic	Refusal	PRR[1]	95% CI[1]	p-value	Refusal	PRR[1]	95% CI[1]	p-value
Yes	29	1.06	0.68, 1.60	0.79	26	1.08	0.66, 1.73	0.74
Mass media								
No	30	—	—		25	—	—	
Yes	83	0.92	0.61, 1.42	0.69	49	0.78	0.48, 1.28	0.30
Health care workers								
No	89	—	—		52	—	—	
Yes	24	0.69	0.43, 1.07	0.11	22	0.60	0.36, 0.97	0.043
Family/friends/neighbors								
No	79	—	—		51	—	—	
Yes	34	1.16	0.77, 1.72	0.46	23	0.94	0.56, 1.52	0.80
Church								
No	104	—	—		51	—	—	
Yes	9	1.15	0.54, 2.14	0.69	23	1.04	0.63, 1.69	0.87
Others								
No	107	—	—		71	—	—	
Yes	6	1.09	0.42, 2.26	0.85	3	2.30	0.56, 6.17	0.16
Trusted source of COVID-19 information								
Mass media	36	—	—	—	59	—	—	
Social media	9	1.51	0.68, 3.01	0.27	0	0.00	0.00, Inf	0.99
Health Care providers	31	1.00	0.61, 1.61	0.99	7	0.70	0.32, 1.53	0.37
Family-members	6	1.56	0.59, 3.44	0.31	3	2.17	0.68, 6.93	0.19
I do not trust any source	15	2.15	1.14, 3.84	0.013	2	1.90	0.31, 5.91	0.37
Government	15	1.05	0.56, 1.87	0.88	3	0.62	0.19, 1.98	0.42
Others	1	0.72	0.04, 3.30	0.74	2	2.90	0.71, 11.9	0.14
COVID-19 knowledge								
Vaccine protects against infection								
No/Don't know	52	—	—		32	—	—	
Yes	59	0.58	0.40, 0.85	0.005	42	0.56	0.35, 0.89	0.012
Vaccine protects the unvaccinated								
Yes	37	—	—		22	—	—	
No/ Don't know	76	1.43	0.97, 2.14	0.074	52	1.28	0.79, 2.15	0.33
Children can be vaccinated								

	Urban n=122				Rural n=77			
Characteristic	Refusal	PRR[1]	95% CI[1]	p-value	Refusal	PRR[1]	95% CI[1]	p-value
No/ Don't know	73	—	—		55	—	—	
Yes	40	0.90	0.61, 1.31	0.58	19	0.60	0.35, 0.99	0.056
Vaccine has no side effects								
No/ Don't know	80	—	—		57	—	—	
Yes	33	1.20	0.79, 1.78	0.38	17	0.75	0.42, 1.26	0.30
COVID-19 vaccine attitudes								
It's important to get a COVID-19 vaccine								
Strongly Agree/Agree	83	—	—		62	—	—	
Neutral	18	1.96	1.14, 3.18	0.010	9	1.83	0.85, 3.50	0.089
Strongly Disagree/Disagree	12	2.52	1.31, 4.43	0.003	3	2.44	0.60, 6.58	0.13
Government should avail vaccines to everyone								
Strongly Agree/Agree	95	—	—		60	—	—	
Strongly Disagree/Disagree	8	2.19	0.98, 4.22	0.033	5	2.71	0.95, 6.10	0.032
Neutral	10	1.82	0.89, 3.33	0.070	9	1.39	0.64, 2.66	0.35
I am willing to pay for the vaccine								
Neutral	10	—	—		9	—	—	
Strongly Disagree/Disagree	87	1.26	0.69, 2.58	0.49	61	1.16	0.61, 2.51	0.67
Strongly Agree/Agree	16	0.61	0.28, 1.39	0.22	4	0.54	0.15, 1.65	0.30
I trust the COVID-19 vaccine information from the media								
Neutral	17	—	—		15	—	—	
Strongly Agree/Agree	75	0.97	0.59, 1.69	0.90	56	0.47	0.27, 0.86	0.009
Strongly Disagree/Disagree	21	1.59	0.84, 3.05	0.16	3	0.80	0.19, 2.42	0.72

PRR[1] = Prevalence Rate Ratio, CI[1] = Confidence Interval
